# Efficacy and safety of transcutaneous electrical acupoint stimulation for preoperative anxiety in thoracoscopic surgery: a randomized controlled trial

**DOI:** 10.3389/fmed.2025.1527993

**Published:** 2025-04-07

**Authors:** Jie Zhang, Xindi Wu, Chenni Ju, Subinuer Kurexi, Xiaoxiao Zhou, Ke Wang, Tongyu Chen

**Affiliations:** ^1^Yueyang Hospital of Integrated Traditional Chinese and Western Medicine, Shanghai University of Traditional Chinese Medicine, Shanghai, China; ^2^Shanghai University of Traditional Chinese Medicine, Shanghai, China; ^3^Yueyang Hospital of Integrated Traditional Chinese and Western Medicine, Acupuncture Anesthesia Clinical Research Institute, Shanghai University of Traditional Chinese Medicine, Shanghai, China

**Keywords:** electrical acupoint stimulation, preoperative anxiety, general anesthesia, video-assisted thoracoscopic surgery, randomized trial, peri-operative

## Abstract

**Background:**

Patients undergoing video-assisted thoracoscopic surgery (VATS) often experience preoperative anxiety, which can significantly impact the surgical process and postoperative recovery. However, the efficacy of Transcutaneous Electrical Acupoint Stimulation (TEAS) in managing preoperative anxiety in VATS patients is unknown.

**Methods:**

A total of 82 patients scheduled for thoracoscopic surgery were randomly divided into TEAS group (*n* = 41) and sham TEAS (STEAS) group (*n* = 41). The TEAS/STEAS intervention began 3 days before the thoracoscopic surgery, with one session lasting 30 min per day for three consecutive days. The primary outcome measure will be the change in Generalized Anxiety Disorder Scale scores between the day before surgery and the baseline. Secondary outcome include intraoperative anesthetic consumption, time to postoperative chest tube removal, postoperative analgesic consumption and pain scores, length of postoperative hospital stay, serum concentrations of 5-hydroxytryptamine (5-HT), norepinephrine (NE), and gamma-aminobutyric acid (GABA).

**Results:**

On the third intervention day, anxiety levels in the TEAS group were significantly lower than in the STEAS group (*p* < 0.01). TEAS patients required less intraoperative sufentanil, remifentanil, and dexamethasone (*p* < 0.01). Chest tube removal time and hospital stay were shorter in the TEAS group (*p* < 0.01). Postoperative meperidine consumption and VAS pain scores were lower in the TEAS group (*p* < 0.01). Serum 5-HT levels were lower in the TEAS group on day three (*p* < 0.01), while NE levels remained lower from day three of intervention to postoperative day three (*p* < 0.05). GABA levels were higher in the TEAS group (*p* < 0.01).

**Conclusion:**

TEAS effectively reduces preoperative anxiety, decreases intraoperative anesthetic and anti-inflammatory drug use, shortens postoperative chest tube removal time and hospitalization, and alleviates postoperative pain. These results indicate that TEAS, as an adjunctive therapy, has valuable potential in improving surgical outcomes and postoperative experience for patients with pulmonary nodules.

**Clinical trial registration:**

https://clinicaltrials.gov, NCT04887090.

## 1 Introduction

Preoperative anxiety is generally described as a state of discomfort or tension caused by disease, the ward environment, or worries about anesthesia and surgery before the operation; this state is often accompanied by somatic manifestations such as palpitations, dyspnea, sweating, frequent urination, abdominal pain, diarrhea or sleep disturbance (1). According to the World Health Organization (WHO), between 266 and 360 million surgical procedures are performed annually worldwide (2). It is estimated that over 50% of patients will experience some level of preoperative anxiety (3). It not only affects the psychological well-being of patients but also induces changes in their vital signs, thereby influencing the surgical and anesthetic processes. This, in turn, escalates the incidence of postoperative complications, particularly in severe cases. Furthermore, preoperative anxiety can heighten postoperative pain sensitivity, suppress immune function, increase the likelihood of postoperative infection, and prolong the postoperative recovery period (4). While the level of preoperative anxiety varies depending on the type of surgery (5), 34% of patients referred for thoracic surgery require psychological intervention and are more likely to develop an increased level of anxiety (6). Anxiety before surgery not only increases the risk and difficulty of the management of surgery and anesthesia, but also causes persistent anxiety after surgery, increases postoperative sensitivity to pain, suppresses immune function, increases postoperative infection, and prolongs postoperative recovery time. Even common minimally invasive procedures like VATS can lead to excessive complications and pathophysiological changes due to preoperative anxiety, ultimately affecting patients’ postoperative experiences and prognostic outcomes (7).

For preoperative anxiety, the main clinical use of psychotherapy and drug therapy. Psychotherapy, while effective, demands a significant amount of time, incurs high costs, and may have limited applicability in the preoperative setting. As pharmacological treatments have adverse effects such as breathing problems, drowsiness, interfering with anesthetic drugs, and prolonged recovery, non-pharmacological interventions are becoming more commonly used. It is reported that non-pharmacological interventions are more commonly used by anesthesiologists compared to pharmacologic ones in both pediatric and adult anesthesia procedures (8). Prior research has demonstrated the effectiveness of acupuncture in alleviating preoperative anxiety and promoting postoperative recovery, without the common side effects associated with pharmaceuticals (9).

TEAS is a contemporary therapy derived from traditional acupuncture, utilizing electrical pulses delivered to acu-points via surface skin electrodes (10). Compared to acupuncture, TEAS involves placing surface electrodes on acupuncture points, allowing the current to stimulate the acupoint area through the skin. This method avoids the invasive procedure of needle insertion, offering several advantages, including being non-invasive, high acceptability, extremely low infection rates, ease of repetition, and low cost. Most trials have primarily concentrated on perioperative analgesia, gastrointestinal function adjustment, and early recovery with TEAS (11). Based on previous research and clinical experience, acupuncture has been shown to improve mental states and alleviate postoperative pain (12). Consequently, TEAS therapy can be a beneficial intervention in the management of preoperative anxiety.

Although clinical studies have established that acupuncture is an effective method for postoperative pain control and anxiety reduction (13), there is limited evidence regarding the effectiveness and safety of TEAS in managing preoperative anxiety in patients undergoing thoracoscopic surgery. Thus, we have designed a prospective pilot randomized controlled trial comparing TEAS and STEAS to acquire more robust evidence supporting the efficacy and safety of TEAS in the preoperative management of anxiety before thoracoscopic surgery.

## 2 Materials and methods

### 2.1 General materials

**Figure 1:**
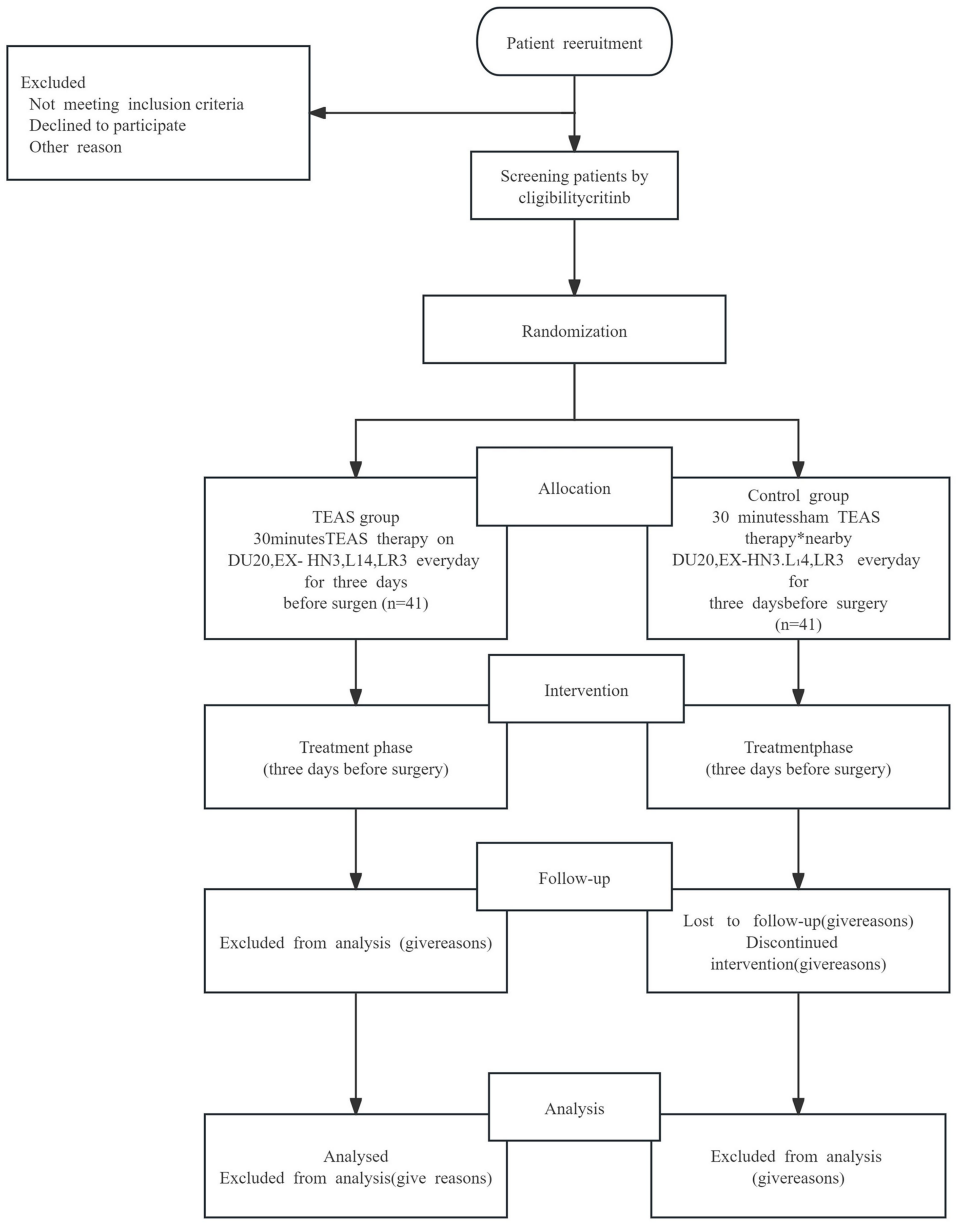
Study flow chart. Transcutaneous electrical acupoints stimulation. Sham TEAS therapy: The acupoints selection and intervention measures are the same as the TEAS group except for the current intensity is set to 0 mA. TEAS, transcutaneous electrical acupoints stimulation.

A total of 82 eligible participants with pulmonary nodules (size ≥8 mm) at Yueyang Hospital of Integrated Traditional Chinese and Western Medicine, affiliated with Shanghai University of Traditional Chinese Medicine, from late June 2021 to March 2023, were randomly allocated to either the TEAS group (n = 41) or the STEAS group (*n* = 41). This study received ethical approval from the Ethics Committee of Yueyang Hospital of Integrated Traditional Chinese and Western Medicine, affiliated with Shanghai University of Traditional Chinese Medicine (approval number: 2021–023). This trial protocol has been registered on ClinicalTrials.gov PRS^1^ with the registration number: NCT04887090. Informed consent was obtained from all participants. The study flowchart is presented in Figure 1.

### 2.2 Inclusion criteria

Patients were included if they met the diagnosed as pulmonary nodules requiring VATS (14); were aged 18–75 years old (either sex); 4 ≤ generalized Anxiety Disorder-7 (GAD-7) score ≤ 9 and have no previous mental illness and no use of anti-anxiety medications or psychotropic drugs within 2 weeks (15); were fully conscious; have not previously received TEAS treatment; agreed to sign an informed consent form; were not enrolled in or were participating in other clinical trials 1 month prior to the enrolment.

### 2.3 Exclusion criteria

Patients with local acupoint skin infection; upper or lower limb nerve injury; who with implanted pacemaker.

### 2.4 Randomization and blinding

Randomization was carried out using IBM SPSS Statistics Version 21.0 software. The group assignments, performed in a randomized sequence, were placed inside sealed confidential envelopes. Eligible participants were then randomized in a 1: 1 ratio to either the TEAS group or the STEAS group.

To ensure the integrity of the blinding process, all researchers involved in the study, with the exception of the acupuncturists, remained blinded to the group assignments. Additionally, both efficacy evaluators and statisticians were kept separate and also remained blinded throughout the study.

In order to assess the effectiveness of participant blinding, all participants were asked to guess whether they had received TEAS or STEAS within 5 min after one of the treatment sessions preceding VATS.

### 2.5 Rejection, suspension and dropout criteria

Participants experiencing serious adverse reactions; Individuals with worsening symptoms or life-threatening illnesses that make it impossible to continue treatment; Identification by the principal investigator of an unacceptable risk of serious adverse events (AEs) during the study period; Patients who are unable to complete the study plan due to factors such as speech difficulties, infectious diseases, or other medical history issues; Participants who voluntarily withdraw from the study.

### 2.6 Standard treatment protocol

According to the consensus on VATS (Video-Assisted Thoracic Surgery) pulmonary resection in thoracic surgery, each patient underwent the same standardized surgical protocol and perioperative management. All VATS procedures in our study were performed using a single port approach. Except for TEAS, other perioperative adjunctive treatments, including benzodiazepines and other anxiolytic sedatives, cognitive behavioral therapy, music therapy, and massage therapy, were not permitted during the study period.

Intraoperative anesthesia included a loading dose of dexmedetomidine at 1 μg/kg for 10–15 min, followed by a continuous infusion of 0.5 μg/kg/h after the initial injection, with a concentration of 4 μg/mL. Small doses of sufentanil (5 μg) were administered in divided doses intravenously, with a typical dose of 10–15 μg; remifentanil was given at a dose of 0.5–1 μg/kg in intervals via intravenous bolus. The depth of sedation was adjusted according to the Ramsay Sedation Scale, with a target score of 3, meaning the patient was sedated but still responsive to commands; the BIS (Bispectral Index) was maintained between 60 and 80 to avoid excessive sedation affecting respiration.

If the intraoperative respiratory rate was too high, it was controlled by additional doses of 5 μg of sufentanil or by adjusting the target concentration of remifentanil to achieve smooth respiratory motion with tidal volume of 3–5 mL/kg, respiratory rate of 8–15 bpm, and stable PaCO2 levels. If the patient experienced persistent hypoxemia, severe dyspnea, frequent coughing, inadequate pain control, or hemodynamic instability, conversion to general anesthesia and endotracheal intubation should be considered. Additionally, if dense adhesions, massive bleeding, or anatomical abnormalities were discovered during the surgery, conversion to general anesthesia and endotracheal intubation should also be considered.

At the end of the thoracic procedure, after placement of the drainage tube, the patient was instructed to take deep breaths and cough to facilitate lung re-expansion. All anesthetic agents were stopped before suturing the skin, and the patient was allowed to wake further. Pethidine hydrochloride (meperidine) was used as the postoperative analgesic, administered via intramuscular injection at a dose of 0.3 mg/kg initially, with additional doses provided as needed for pain management.

### 2.7 Interventions

TEAS group: In the TEAS group, 30 min of TEAS treatment was performed by an experienced acupuncturist. TEAS was performed at the DU20 (Baihui), EX-HN3 (Yintang) and both sides of LI4 (Hegu), LR3 (Taichong). Starting on 3 days before VATS, by means of a stimulator (HANS200A Beijing Huawei) (16). Additionally, The 30-min treatments were delivered 1 time a day for three consecutive days. The TEAS device was calibrated before each treatment and was set to 2 Hz / 100 Hz for effective anxiety relief (17). The current intensity was adjusted individually, starting at 1 mA and gradually increased to the maximum current tolerated by the patient.

Except for TEAS, other therapies aimed at reducing anxiety levels, such as anxiolytic-sedative drugs like benzodiazepines, cognitive-behavioral therapy, music therapy, and massage therapy, are prohibited during the study period. Additionally, every patient will follow a consistent and standardized surgical protocol and perioperative management (18).

STEAS group: the same acupoints are selected as those in the TEAS group, and other intervention measures are also the same as those in the TEAS group, except that the current intensity is set to 0 mA. The acupuncturist responsible for the operation will tell patients in STEAS group that this is a type of stimulus without perception.

### 2.8 Outcome measures

The primary outcome is the change in GAD-7 scores between the baseline (Day 0; the day of enrollment), the day before surgery (Day 1; 3 days before surgery, prior to TEAS/STEAS treatment), and after the third treatment (Day 3; the day before surgery, after the third TEAS/STEAS treatment). The GAD-7, a seven-item scale designed to assess anxiety disorders (where higher scores indicate more severe anxiety symptoms), will be used for this assessment (19).

The secondary outcomes included intraoperative anesthetic consumption, time to postoperative chest tube removal, postoperative pain, length of postoperative hospital stay, and 5-hydroxytryptamine (5-HT), norepinephrine (NE), and gamma-aminobutyric acid (GABA). Detection method: Peripheral venous blood was collected from patients 3 days before surgery, 1 day before surgery, on postoperative Day 1, and on postoperative Day 2, after TEAS treatment. The blood was centrifuged to separate the serum and stored at −80°C until analysis. The serum concentrations of 5-HT, NE, and GABA were measured using enzyme-linked immunosorbent assay (ELISA). The total amount of pethidine hydrochloride used for postoperative pain relief was recorded within 72 h after surgery. Postoperative pain was then assessed daily for 2 days following VATS using the Visual Analog Scale (VAS), ranging from 0 (no pain) to 10 (unbearable pain). Additionally, the length of the postoperative hospital stay was recorded.

### 2.9 Safety outcomes

Adverse reactions associated with TEAS, particularly skin itching, rash, or swelling related to the electrodes, as well as mood disturbances, will be vigilantly monitored. This monitoring will occur through spontaneous reports by participants, clinical observations by acupuncturists and surgeons, or by direct inquiries to participants with open-ended questions. In the event of any adverse reactions, TEAS/STEAS administration will be promptly discontinued. Additionally, appropriate corrective measures will be implemented as necessary. If required, participants will be referred for expectant treatment of the symptoms.

Detailed records will be maintained in the electronic case report form (eCRF) regarding the time of occurrence, duration, severity of symptoms, potential causes, and the corresponding management of adverse events (AEs).

In cases of severe AEs, the ethics committee will be promptly notified.

### 2.10 Sample size

Primary outcome indicator in this trial is the GAD-7 score variation between the day before VATS with the baseline. The sample size was calculated on the grounds of our preliminary trial (online supplemental material 1). Reference for optimal effectiveness in clinical trials of two sample mean comparison, using formula for calculating sample size of clinical trials as follow.

GAD-7 score change in TEAS group increase the divergence between the STEAS group and the SD by 73%. *δ*/*σ* = 0.73, test level bilateral *α* = 0.05, test power 1 − *β* = 0.9. Through calculation, each group required sample size is 40 samples. The submitted trial will demand 82 participants to be divided into two groups of 41 in consideration of the 15% probability of lost to follow-up.

### 2.11 Statistical analysis

Blinded independent statisticians will analyze the data using IBM SPSS Statistics Version 21.0. Continuous variables will be presented as Mean ± SD for normally distributed data and as median with IQR for skewed data. Categorical variables will be represented as percentages and analyzed with the χ^2^ test.

Inter-group differences will be assessed with Independent two-sample t-tests, while intra-group comparisons will use paired t-tests for normally distributed variables and the Mann–Whitney rank sum test for skewed variables. Generalized linear models will be employed for repeated measurements of continuous variables.

Statistical significance will be defined as *p* < 0.05 (two-sided, 95% CI), and Kappa analysis will determine whether participants correctly guessed their group allocation.

## 3 Results

### 3.1 Baseline characteristics

Based on the intention-to-treat (ITT) analysis, there were no statistically significant differences in baseline characteristics between the two groups, indicating comparability. The demographic, clinical, and surgical characteristics of the patients are summarized in Table 1.

**Table 1:** Baseline characteristics.

Project	TEAS (*n* = 41)	STEAS (*n* = 41)	p
Age (yr)	59.63 ± 11.56	61.83 ± 9.03	0.34
Female	26 (63.42)	19 (63.41)	0.12
Male	15 (36.58)	22 (36.59)	
BMI (kg/m2)	23.55 ± 3.77	23.42 ± 3.08	0.86
FEV1 (L)	2.28 ± 0.75	2.33 ± 0.62	0.70
FEV1/FVC (%)	80.07 ± 10.74	80.45 ± 10.18	0.87
GAD-7	4.76 ± 1.04	4.39 ± 0.66	0.62
Surgical Time (min)	94.27 ± 35.79	101.07 ± 28.31	0.34
Cerebral oxygen saturation	98.83 ± 1.18	98.85 ± 1.51	0.93
PaO₂	104.85 ± 35.50	105.68 ± 23.79	0.90
PaCO₂	42.07 ± 3.94	43.15 ± 4.97	0.28
Wedge resection of the lung	15 (36.58)	13 (31.71)	0.90
egmental Resection of the Lung	13 (31.71)	14 (34.14)	
Lobectomy	13 (31.71)	14 (34.14)	
Hypertension	12 (29.27)	13 (31.71)	1.59
Diabetes mellitus	4 (9.76)	3 (7.32)	
Coronary artery disease	2 (4.88)	3 (7.32)	
Coronary artery disease	2 (4.88)	3 (7.32)	

BMI (kg/m^2^): Body Mass Index, calculated as weight (kg) divided by height (m^2^).FEV₁ (L): Forced Expiratory Volume in 1 s, measured in liters. FEV₁/FVC (%): Ratio of Forced Expiratory Volume in 1 s (FEV₁) to Forced Vital Capacity (FVC), expressed as a percentage. Cerebral Oxygen Saturation: The oxygen saturation level in the brain, measured by near-infrared spectroscopy. PaO₂: Partial pressure of oxygen in arterial blood, measured in mmHg. PaCO₂: Partial pressure of carbon dioxide in arterial blood, measured in mmHg. Comparison of baseline demographic and clinical characteristics [*n* (%), Mean ± SD].

### 3.2 Generalized anxiety disorder scale scores

On Day 0 (baseline), there was no statistically significant difference between the groups, with GAD-7 scores (*p* = 0.62). By Day 1, the GAD-7 scores had increased slightly in both groups, but was no statistically significant difference (*p* = 0.10). However, by Day 3, the TEAS group showed a marked reduction in GAD-7 scores, with the statistically significant difference (*p* < 0.01; Table 2).

**Table 2:** Generalized anxiety disorder scale scores.

Time	TEAS (*n* = 41)	STEAS (*n* = 41)	p
Day 0	4.76 ± 1.04	4.39 ± 0.66	0.62
Day 1	6.95 ± 1.28	6.54 ± 1.00	0.10
Day 3	2.73 ± 1.62	6.71 ± 1.25	<0.01^**^

Day 0: Baseline (the day of enrollment); Day 1: Pre-intervention (3 days before surgery, prior to TEAS/STEAS treatment); Day 3: Post-third treatment (the day before surgery, after the third TEAS/STEAS treatment).TEAS: Electrical Acupoint Stimulation, STEAS: Sham TEAS. Comparison of GAD-7 Scores between Two Groups (mean ± SD).

### 3.3 Intraoperative anesthetic consumption

No significant difference was observed in the dexmedetomidine (Dex) dose administered before anesthesia induction between the TEAS and STEAS groups (*p* = 0.69). However, during surgery, the TEAS group required significantly lower doses of Dex (*p* < 0.01), remifentanil (*p* < 0.01), and sufentanil (*p* < 0.01; Table 3) compared to the STEAS group.

**Table 3:** Intraoperative anesthetic consumption.

Observation index	TEAS (*n* = 41)	STEAS (*n* = 41)	p
Dex before induction of anesthesia	63.89 ± 11.91	64.87 ± 10.47	0.69
Dex	77.72 ± 25.88	245.97 ± 73.41	<0.01
Remifentanil	509.14 ± 261.17	707.92 ± 334.87	<0.01
Sufentanil	13.59 ± 5.35	18.47 ± 8.53	<0.01

TEAS, Electrical acupoint stimulation; STEAS, Sham TEAS. Comparison of intraoperative anesthetic dosage between two groups (μg, mean ± SD).

### 3.4 Postoperative analgesic use and pain score assessment

The TEAS group showed a significant reduction in meperidine consumption within 72 h postoperatively compared to the STEAS group (*p* < 0.01; Table 4).

**Table 4:** Postoperative analgesic use and pain score assessment.

Observation index	TEAS (*n* = 41)	STEAS (*n* = 41)	p
Pethidine hydrochloride(mg)	24.39 ± 38.52	54.88 ± 46.17	<0.01
Postoperative VAS scores			
Postoperative 1 h	5.37 ± 1.01	5.20 ± 0.95	0.43
Postoperative 2 h	3.76 ± 1.65	6.02 ± 1.31	<0.01
Postoperative 24 h	2.56 ± 1.00	5.27 ± 1.26	<0.01
Postoperative 48 h	0.85 ± 0.98	2.8 ± 0.90	<0.01
Postoperative 72 h	0.29 ± 0.51	2.02 ± 0.75	<0.01
Time to chest tube removal after surgery(h)	52.24 ± 20.60	98.20 ± 43.17	<0.01
Postoperative hospital stay (days)	6.37 ± 1.62	8.73 ± 2.46	<0.01

TEAS Electrical Acupoint Stimulation, STEAS sham TEAS. Comparison of Piperidine Hydrochloride Usage and Postoperative VAS Scores between TEAS and STEAS Groups (mean ± SD).

From 2 h postoperatively onward, the TEAS group had significantly lower VAS scores compared to the STEAS group (*p* < 0.01; Table 4).

### 3.5 Chest tube removal time and postoperative hospital stay duration

The time to chest tube removal after surgery was significantly shorter in the TEAS group compared to the STEAS group (*p* < 0.01; Table 4).

Postoperative hospital stay was significantly shorter in the TEAS group compared to the STEAS group (*p* < 0.01; Table 4).

### 3.6 Neurotransmitter indicators

On Day 1, there was no statistically significant difference in serum 5-HT concentrations between the TEAS and STEAS groups (*p* = 0.56). However, by Day 3, the TEAS group exhibited significantly lower 5-HT levels compared to the STEAS group (*p* < 0.01). Although a trend toward lower 5-HT levels persisted in the TEAS group on Day 5, the difference was no significant difference (*p* = 0.06). On Day 6, there was no significant difference between the two groups (*p* = 0.44; Table 5).

**Table 5:** Serum neurotransmitters concentration between two groups (ng/mL, mean ± SD).

Neurotransmitter	Time	TEAS (*n* = 41)	STEAS (*n* = 41)	p
5-HT	Day 1	371.56 ± 337.74	361.72 ± 324.98	0.56
	Day 3	195.27 ± 182.76	331.24 ± 297.31	<0.01
	Day 5	235.09 ± 223.45	324.09 ± 305.27	0.06
	Day 6	279.10 ± 266.60	312.28 ± 300.79	0.44
NE	Day 1	5.01 ± 3.15	5.17 ± 4.02	0.45
	Day 3	4.17 ± 2.03	7.40 ± 5.22	0.02
	Day 5	4.59 ± 2.16	7.31 ± 5.16	0.03
	Day 6	4.32 ± 2.21	6.81 ± 5.04	0.02
GABA	Day 1	2.62 ± 2.20	2.34 ± 2.00	0.71
	Day 3	42.58 ± 38.17	3.91 ± 3.17	<0.01
	Day 5	37.63 ± 34.89	3.50 ± 2.79	<0.01
	Day 6	39.13 ± 33.54	8.45 ± 4.99	<0.01

Day 1: Three days before surgery, prior to TEAS/STEAS intervention; Day 3: One day before surgery, after the third TEAS/STEAS intervention; Day 5: Postoperative Day 1, after TEAS/STEAS intervention; Day 6: Postoperative Day 2, after TEAS/STEAS intervention. TEAS Electrical Acupoint Stimulation, STEAS sham TEAS, 5-HT 5-hydroxytryptamine, NE norepinephrine, GABA gamma-aminobutyric acid. Statistical significance was defined as *p* < 0.05.

No significant difference was found in serum NE concentrations on Day 1 between the TEAS and STEAS groups (*p* = 0.45). By Day 3, NE levels were significantly lower in the TEAS group compared to the STEAS group (*p* = 0.02). This significant reduction in NE concentration in the TEAS group persisted through Day 5 (*p* = 0.03) and Day 6 (*p* = 0.02; Table 5).

On Day 1, no significant difference in serum GABA concentrations was observed between the TEAS and STEAS groups (*p* = 0.71). However, by Day 3, the TEAS group showed a significantly higher GABA concentration than the STEAS group (*p* < 0.01). This difference remained significant on Day 5 (*p* < 0.01) and Day 6 (*p* < 0.01; Table 5).

## 4 Discussion

This study explored the effects of TEAS on preoperative anxiety in patients undergoing thoracoscopic surgery. The results showed that TEAS not only significantly reduced preoperative anxiety but also decreased intraoperative anesthetic consumption and postoperative pain, promoting patient recovery. Compared to the STEAS group, the GAD-7 scores in the TEAS group were significantly lower. Additionally, no serious adverse events or safety concerns related to TEAS were observed, indicating that TEAS is a safe, effective, and clinically acceptable method for managing preoperative anxiety.

Preoperative anxiety is characterized by restlessness and tension, often accompanied by abnormal activation of the autonomic nervous system (20). Research indicates that over 70% of surgical patients experience some level of preoperative anxiety (21). In this study, TEAS significantly improved patients’ anxiety levels, consistent with the findings of previous research (22).

When preoperative anxiety is accompanied by significant stress, it can lead to postoperative complications, prolonged hospital stays, delayed recovery, and increased reliance on higher doses of anesthetics and pain medications (23). Our study found that TEAS reduced intraoperative anesthetic consumption, shortened the time required for chest tube removal, and alleviated postoperative pain. Previous studies have also shown that TEAS can effectively reduce postoperative pain by lowering local pain thresholds and relieving discomfort (24). TEAS has been demonstrated to alleviate postoperative symptoms, which aligns with the findings of other studies (25).

For postoperative pain, studies have shown that electroacupuncture can upregulate endocannabinoid levels, directly inhibiting pain by activating cannabinoid receptor 2, thereby reducing sensory nerve activity, as observed in a rat pain model (26). Similarly, TEAS has been demonstrated to produce comparable therapeutic effects to electroacupuncture (27). Second, electroacupuncture at the LI4 (Hegu) acupoint has been shown to alter the phosphorylation level of the N-methyl-D-aspartate (NMDA) receptor subunit 2B in the C1–C3 segment of the spinal cord, while also upregulating the expression of 5-hydroxytryptamine 2A receptor mRNA and protei, which could effectively increase the pain threshold and relieve pain (28). Furthermore, experimental studies have demonstrated that alternating between low (2 Hz) and high (100 Hz) frequency stimulation induces a stronger release of endorphins in the central nervous system compared to single-frequency stimulation (29). Clinical practice has confirmed that alternating low and high frequencies enhances the analgesic effect (30). Therefore, we have chosen to use both 2 Hz and 100 Hz frequencies in our study to ensure the therapeutic efficacy.

Moreover, the TEAS group exhibited significantly shorter chest tube removal times and postoperative hospital stays. This outcome serves as further evidence that TEAS can facilitate postoperative recovery. An RCT showed that TAES treatment can increase the serum levels of IL-2 and IFN-c, and decreased IL-4 secretion and return the aforementioned cellular immune factors to the preoperative control value at a faster rate (31). There is research evidence to suggest that TAES can alleviate postoperative immune dysfunction by modulating the expression of TH1/TH2 cell-associated cytokines (32). TEAS can promote gastrointestinal motility and reduce the occurrence of nausea and vomiting through the stimulation of 5-HT and norepinephrine fibers (33). This can help mitigate the side effects associated with opioid medications. This provides a reference for studying the mechanism of TEAS in promoting postoperative recovery.

HT, NE, and GABA are all considered closely related to the pathophysiology of anxiety and exhibit interconnections in terms of neurobiology (34). Patients with anxiety often demonstrate dysregulation of these neurotransmitters, and treatments targeting the 5-HT system may directly or indirectly affect other neurotransmitter systems, including GABA and NE. Our findings indicate that TEAS can reduce preoperative GAD-7 scores, decrease the concentrations of 5-HT and NE, and increase GABA levels, thereby demonstrating that preoperative TEAS treatment can alleviate preoperative anxiety.

EA enhances 5-HT function by reducing HPA axis hyperactivity, lowering cortisol (CORT) levels, and promoting 5-HT synthesis and release (35). Additionally, EA may upregulate 5-HT receptors (5-HT1A, 5-HT1B), enhancing 5-HT-mediated neurotransmission, which helps improve mood (36).

For NE regulation, EA inhibits sympathetic nervous activity and enhances vagal nerve function, reducing NE secretion from sympathetic nerve terminals. EA also downregulates the SAM axis, lowering NE and epinephrine release, while reducing CORT levels to indirectly suppress NE overactivation (37).

EA also enhances GABAergic activity by upregulating glutamate decarboxylase (GAD65 and GAD67), increasing GABA levels, and restoring inhibitory neurotransmission, contributing to its anxiolytic and antidepressant effects (38).

These findings suggest that EA balances excitatory and inhibitory neurotransmission through the coordinated regulation of 5-HT, NE, and GABA systems, alleviating stress-related dysfunction and stabilizing emotional states.

It is noteworthy that some limitations remain in this study. First, the study only included a sample from a single center and may have some selection bias. Secondly, this study only observed the short-term effect after the intervention, the follow-up time is relatively short, and the long-term effect needs further study.

## 5 Conclusion

TEAS can effectively reduce preoperative anxiety in patients undergoing thoracoscopic surgery, reduce the consumption of anesthesia and anti-inflammatory drugs during surgery, significantly reduce postoperative pain, and accelerate the recovery of patients. Furthermore, no serious adverse events related to TEAS and the occurrence of treatment-related safety issues were reported in the included studies. These findings suggest that TEAS, as an adjuvant therapy, is relatively safe and effective in clinical applications.

## Data Availability

The raw data supporting the conclusions of this article will be made available by the authors, without undue reservation.

## Footnotes

^1^ https://clinicaltrials.gov
